# Reactive Nanofiller Reinforced Hybrid Polyurea: The Role of CNC in Material Preparation and Characterization

**DOI:** 10.3390/polym17111527

**Published:** 2025-05-30

**Authors:** Kadir Duman, Madalina Ioana Necolau, Elena Iuliana Bîru, Anamaria Zaharia, Horia Iovu

**Affiliations:** 1 Advanced Polymer Materials Group, National University of Science and Technology Politehnica Bucharest, 1–7 Gh. Polizu Street, 011061 Bucharest, Romania; kadir.duman@stud.chimie.upb.ro (K.D.);; 2 Academy of Romanian Scientists, Ilfov 3, 050044 Bucharest, Romania; 3 Advanced Polymer Materials and Polymer Recycling Group, National Institute for Research and Development in Chemistry and Petrochemistry-ICECHIM, Splaiul Independenței 202, 060021 Bucharest, Romania

**Keywords:** cellulose nanocrystals, polyurea nanocomposite, functionalized nanocellulose

## Abstract

This study presents the development and analysis of hybrid polyurea composite materials. Neat polyurea was reinforced with cellulose nanocrystals (CNCs) and isocyanate-modified CNCs (CNC-ISOs) via a two-step prepolymer process. Introducing CNC considerably increased the mechanical strength and stiffness of the polyurea matrix. The tensile strength increased by up to 16.4%, and the Young modulus improved by approximately 29% compared to the pure polyurea. When CNC was functionalized with isocyanate, the interfacial bonding was further improved, and superior dispersion and load transfer were achieved. At 1.5% CNC-ISO loading, the modulus increased by approximately 128% compared to the unmodified matrix. Comprehensive analyses using FT-IR, XPS, DSC, TGA, DMA, tensile testing, and SEM showed that CNC-ISO films not only achieved higher tensile strength and better thermal stability but also formed a denser polymer network as evidenced by the increased crosslinking density. These findings highlight the importance of tailored nanofiller modification to create advanced polyurea composites with enhanced performance suitable for demanding protective and structural applications.

## 1. Introduction

Polyurea is a versatile elastomeric polymer used for the development of advanced protective coatings with a wide range of applicability. It can easily be synthesized by condensation between an isocyanate component (–N=C=O) with a polyamine (NH_2_–R’–NH_2_), thus providing numerous possibilities to tailor the chemical composition and structure of the final material [1,2,3]. Properties such as lightweight, cost-effectiveness, wear resistance, chemical resistance, good mechanical properties, and capacity to absorb impact energy recommend this versatile material in anti-explosive, protection equipment, ballistic and anti-impact applications [4,5,6,7].

Because of its complexity, polyurea inherently behaves as a composite material because its assemblies into a framework incorporating flexible, rubbery segments within rigid regions, thus creating a strong and complex network [8,9,10]. The existence of multiple nitrogen and oxygen atoms in its chemical structure leads to internal hydrogen bonding in soft segments which contributes to the material’s toughness [11,12].

Over the last decade, the use of advanced nanoscale fillers proved to be a viable solution for the improvement in the overall thermal and mechanical properties of polymeric materials, thus considerably expanding the library of potential applications covered by conventional elastomeric materials.

The introduction of various reinforcing agents with different morphologies and dimensions can develop new interactions with the soft domains of polyurea, providing an opportunity to further improve and customize the final performance of the material [13,14]. Up to now, different nanostructures such as graphene oxide [15,16], nano-clays [17,18], silica [19], hydroxyapatite [20], carbon nanotubes [21,22], and nanoparticles [23,24] have been successfully used to synthesize polyurea nanocomposites.

Different dimensions and geometries of nanofillers can have a significant impact on how macromolecular chains re-organize and align in composite formulations. Thus, on the one hand, specific reinforcing agents can improve the segmental mobility of the polymer chains leading to materials that can be more sensitive under high strain rates, while others can restrict segmental mobility leading to high stiffness. Such an improvement is especially beneficial for applications where materials must sustain prolonged high strain rates without irreversible damage.

Cellulose, a natural polysaccharide, is one of the most abundant biopolymers that recently drew researchers’ attention due to its remarkable properties [25]. Cellulose nanocrystal (CNC) is one of the most used forms for the development of composite materials due to its superior mechanical resistance [26,27].

Based on this, the use of cellulose nanocrystals (CNCs) as a reinforcement for polyurea represents an interesting opportunity. Girouard et al.’s experimental studies have shown that the addition of cellulose nanocrystals into polyurethane remarkably improved the mechanical properties of these materials [28]. According to the results, CNC-reinforced polyurethane composites showed more than 200% higher tensile strength and work of fracture compared to the unmodified matrix, without compromising elongation at break. The increment is significant, indicating that CNC can improve the load-bearing capacity and toughness of polyurea. This enhancement is due to the strong hydrogen-bonding interactions that allow for efficient stress transfer between the nanostructured cellulose and the polyurea matrix.

Septevani et al. demonstrated in their study that the incorporation of CNC into rigid polyurethane foam (RPUF) increases compressive strength. This improvement was due to the alignment of CNC along with the foam rise direction [29]. This emphasizes the role of filler orientation in optimizing the mechanical behavior of polymer composites.

Nevertheless, the dispersion of nanoparticles into the polymer matrix is still an issue, and, in most cases, physical or chemical modification is required to be effectively used as a reinforcement factor [30,31,32,33].

Fan et al. [34] demonstrated the development of a supramolecular polyurethane elastomer reinforced with CNC, exhibiting enhanced mechanical strength along with self-healing capabilities and resistance to fatigue, highlighting the multifunctional potential of CNC incorporation. Additionally, Yu et al. [35] provided insights into how the type of cellulose, loading ratio, and fabrication method significantly influence the microstructure and mechanical behavior of polyurethane composites. Nanocrystalline cellulose (CNC) provides superior reinforcement compared to microcrystalline forms, particularly when well dispersed through solution blending or in situ polymerization. These insights highlight the importance of tailoring both the filler morphology and fabrication strategy to optimize interfacial bonding and mechanical strength in polymer–cellulose systems.

Based on the aforementioned studies, this research aims to produce composite materials with improved mechanical performance and thermal stability while addressing sustainability challenges by analyzing the difference between CNC and functionalized CNC in polyurea-based nanocomposite formulations. Isocyanate grafted onto the nanocellulose surface should lead to enhanced interaction with polyurea, producing materials with improved mechanical and thermal properties for more demanding applications.

## 2. Materials and Methods

### 2.1. Materials

Cellulose nanocrystal (CNC) powder (Wide: 10–20 nm, Length: 300–900 nm) in dry form with a moisture content of less than 4.0% was purchased from Nanografi, Ankara, Istanbul, Türkiye. Cellulose nanocrystal (CNC) powder (width 10–20 nm; length 300–900 nm), supplied in dry form (moisture content < 4 wt %), was purchased from Nanografi (Istanbul, Türkiye).

For the functionalization of CNC and for the synthesis of the polyurethane matrix 2,4’ Diphenylmethane Diisocyanate (MDI), LUPRANAT MI was used (BASF, Ludwigshafen, Germany BASF Polyurethanes GmbH, Lemförde, Germany). DESMOPHENE 2061 BD (D-2061) from Bayer served as polyol while the amine part consisted of Jeffamine D2000 (D2000) from Huntsman and ETHACURE 300 (E300) from Albemarle. Apart from that, propylene carbonate was used as a solvent to facilitate the processing, and also, BYK A 530 was chosen as a defoamer whereas Disperbyk 2152 (BYK, Wesel, Germany) was selected as the dispersing agent for optimal processing and composite stability.

Prior to functionalization, CNC powder was dried at 120 °C for 4 h to remove any reabsorbed moisture, ensuring final dried CNC moisture remained below <2%.

### 2.2. Methods

#### 2.2.1. Synthesis of CNC-Grafted Hybrid Polyurea Prepolymer (Part A)

**Figure 1:**
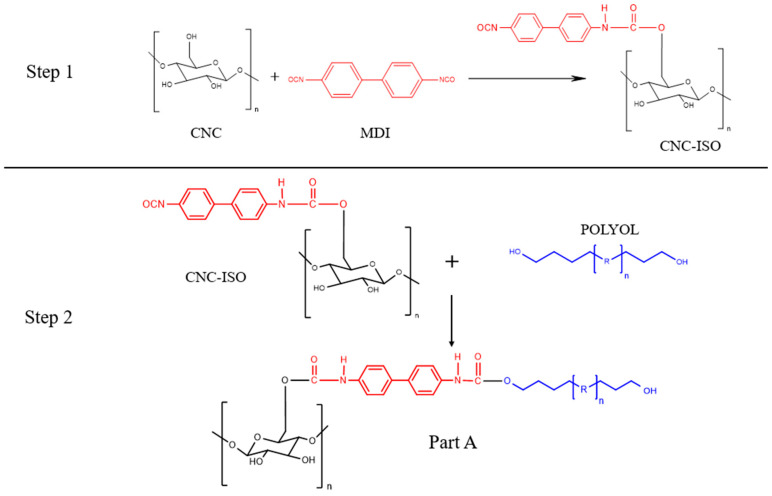
Schematic representation of the main steps involved in the synthesis of CNC-ISO and Part A monomer.

To obtain a hybrid polyurea prepolymer (CNC grafted A part), a two-step protocol was followed. In the first step, monomeric MDI was reacted with CNC. As presented in Figure 1, the hydroxyl (OH) groups of CNC react with isocyanate (NCO) groups of monomeric MDI to form urethane linkages. Further on, the remaining NCO group from the monomeric MDI-CNC complex reacts with the OH group of polyol components, thereby integrating CNC into the prepolymer part of the polyurea.

For that, 2.5 g of completely dried CNC was mixed with 107 g MDI at 80 °C under a nitrogen atmosphere for 4 h. The reaction profile was monitored with FT-IR analysis by tracking the decrease in the characteristic NCO signal. After 4 h of reaction, the mixture was first cooled to room temperature and then was subsequently washed with THF and isopropyl alcohol to remove the unreacted isocyanate. The CNC synthesized in this step will be further described as CNC-ISO.

In the second step of the prepolymer synthesis, CNC-ISO was added to the polyol part in different ratios (0.5%, 1%, and 1.5%) under stirring at 80 °C. In this step, the free isocyanate groups present on the surface of CNC-ISO were reacted with the OH groups from the polyol, yielding the CNC grafted A part. An FTIR analysis was used to monitor this reaction until complete NCO consumption, confirming successful chemical bonding between the CNC-ISO complex and the polyol. After the reaction was complete, the mixture was cooled down and 10% wt. of propylene carbonate was added to dilute the medium. After homogenization, a vacuum was applied to remove the entrapped air. As a reference, neat CNC was added to the polyol prepolymer in different ratios (0.5%, 1%, and 1.5%) by mechanical stirring. Further on, the product obtained from the second step will be generally referred to as Part A. The description of each prepolymer formulation is presented in Table 1 along with the corresponding polyurea matrix and nanocomposites.

#### 2.2.2. Synthesis of Part B

The amine component was synthesized by mechanical stirring between D2000 with E300 at a mass ratio of 6:4 wt. After complete homogenization, a defoamer and dispensing agent were added at 1% by wt. of the total formulation. This process took place at room temperature under a vacuum to prevent oxygen incorporation into the system.

#### 2.2.3. Preparation of CNC-Reinforced Polyurea Films

**Figure 2:**
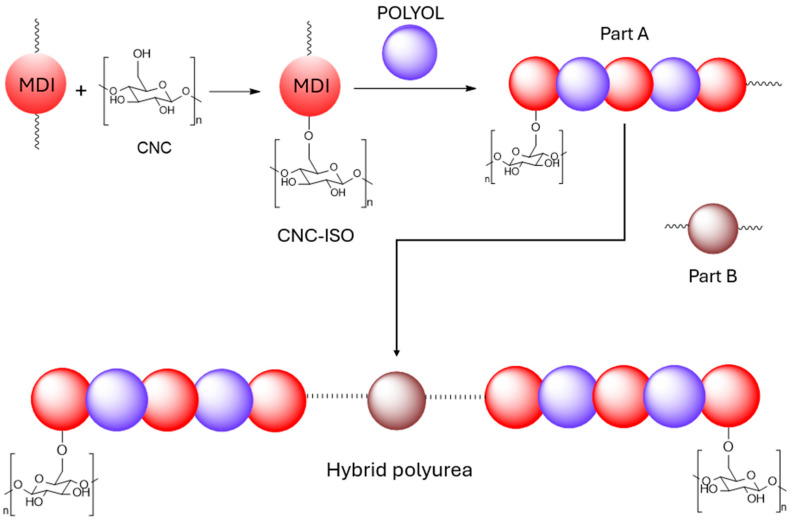
Synthesis mechanism of CNC-ISO Hybrid Polyurea.

The general mechanism involved in the synthesis of hybrid polyurea films is presented in Figure 2. CNC-reinforced polyurea nanocomposites were synthesized by stoichiometrically reacting the different formulations for Part A with the amine component (part B).

**Figure 3:**
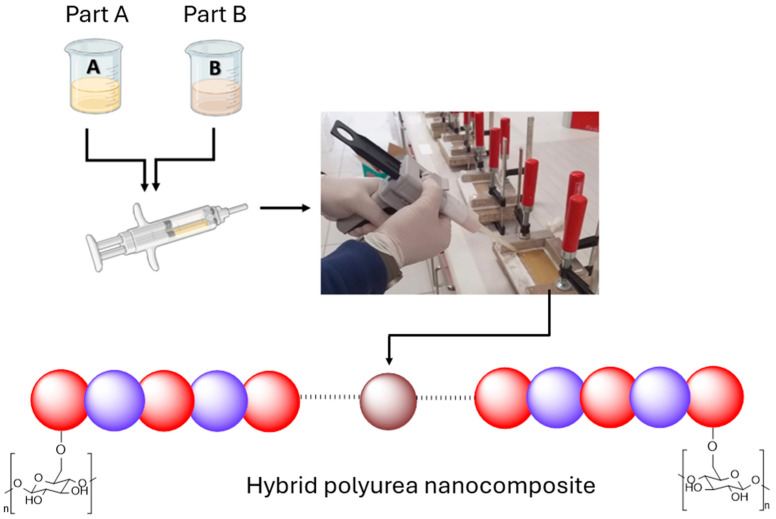
Hybrid polyurea film casting.

Components A and B were loaded into a dual syringe system and cast with a dual gun into Teflon molds (Figure 3). The cast samples were conditioned at 50 °C for 24 h, ensuring complete curing of the polyurea composite. The resulting materials were polyurea nanocomposite films reinforced by CNCs and were denoted PU-CNC (for non-functionalized CNC) and PU-CNC-ISO (for chemically grafted CNC). The films were then evaluated to characterize their mechanical, thermal, and morphological properties.

### 2.3. Characterization

The Fourier Transform Infrared Spectrometry (FTIR) was performed on Bruker VER-TEX 70 equipment (Bruker, Billerica, MA, USA) using 32 scans in the 400–4000 cm^−1^ range, equipped with attenuated total reflection (ATR) using a Ge crystal.

X-ray Photoelectron Spectrometry (XPS) measurements were performed on a Thermo Scientific K-Alpha spectrometer (Thermo Scientific, East Grinstead, UK) with a monochromatic Al Kα source (1486.6 eV) and working in a vacuum base pressure of 2 × 10^−9^ mbar. Charging effects were compensated by a flood gun, and binding energy was calibrated by placing the C1speak at 284.8 eV as the internal standard. The deconvolution of C1s peaks was performed after Shirley background subtraction. The pass energy for the survey spectra was 200 eV, and it was 20 eV for the high-resolution spectra.

Dynamic Mechanical Analysis (DMA) curves were registered on a TRITEC 2000 B device produced by Triton Technology, Ltd. (Baltimore, MD, USA) Now Metler Toledo (Greifensee, Switzerland). The samples were analyzed in the single-cantilever bending mode. The samples were subjected to 1 Hz force and heated in a temperature range from −80 to 0 °C with a heating rate of 5 °C/min.

Equilibrium swelling tests were performed by placing polyurea samples of ~100 mg in toluene at room temperature for 48 h [36]. The swelling degree was computed by weighing the samples after solvent extraction with the aid of the following formula:

(1) $$SD\;\left(\%\right)=\frac{\left(m_{1}-m_{0}\right)}{m_{0}}\times 100$$

where *m*_0_ represents the weight of polyurea samples before swelling and *m*_1_ represents the mass of polyurea samples after swelling.

Crosslinking density was determined by employing the Flory–Rehner equation [37]:

(2) $$CD=-\ln\frac{\left[\left(1-V_{r}\right)+V_{r}+\chi V_{r}^{2}\right]}{\left[V_{s}\left(V_{r}^{\frac{1}{3}}-\frac{V_{r}}{2}\right)\right]}$$

where *V_r_* represents the volume fraction of polyurea samples in the swollen gel, *V_s_* is the molar volume of toluene, and *χ* represents a parameter related to the interaction between polyurea and toluene. *V_r_* (Equation (3)) and *χ* (Equation (4)) were computed with the aid of the following formulas:

(3) $$V_{r}=(\frac{m_{0}}{\rho_{m}})/\left[\frac{m_{0}}{\rho_{m}}+\frac{m_{1}-m_{0}}{\rho_{s}}\right]$$

(4) $$\chi =0.487+0.228\times V_{r}$$

where *ρ_m_* and *ρ_s_* represent the polyurea density and solvent density.

Differential scanning calorimetry (DSC) analyses were performed on Netzsch 204 F1Phoenix equipment (Selb, Germany) by heating the samples in aluminum crucibles from room temperature to 300 °C, in a nitrogen flow rate of 20 mL/min, at a heating rate of 10 °C/min.

A thermogravimetric analysis (TGA) was performed on TA Instruments Q500 equipment (Bellingham, WA, USA) as follows: a sample amount (about 3 mg) was heated in the temperature range of 20–800 °C with a heating rate of 10 °C/min, in air and nitrogen atmosphere, using a platinum crucible.

Mechanical tests were performed by using a universal mechanical tester (Instron, Model 3382, Norwood, MA, USA) at a relative humidity of 45–50% and a speed of 1 mm/min. The size of the samples was approximately 10 × 1 cm. A minimum of three specimens were tested for each polyurea sample, and the average values were reported. Tensile tests were performed by following the European Standard, EN ISO 527-3 Tensile tests were performed by following the European Standard, EN ISO 527-3, Geneva, Switzerland, 2018.

Scanning electron microscopy (SEM) (Spectral, Lidingo, Sweden) analyses were performed on a HitachiTM4000plus II tabletop equipment with a cooling stage and operated at 15 kV. Prior to analysis the samples were fractured and coated with an electrically conductive thin film of gold to inhibit “charging”, reducing thermal damage, and increasing the emission of secondary electrons.

## 3. Results and Discussion

### 3.1. CNC Characterization

#### 3.1.1. Fourier Transform Infrared Analysis (FT-IR)

**Figure 4:**
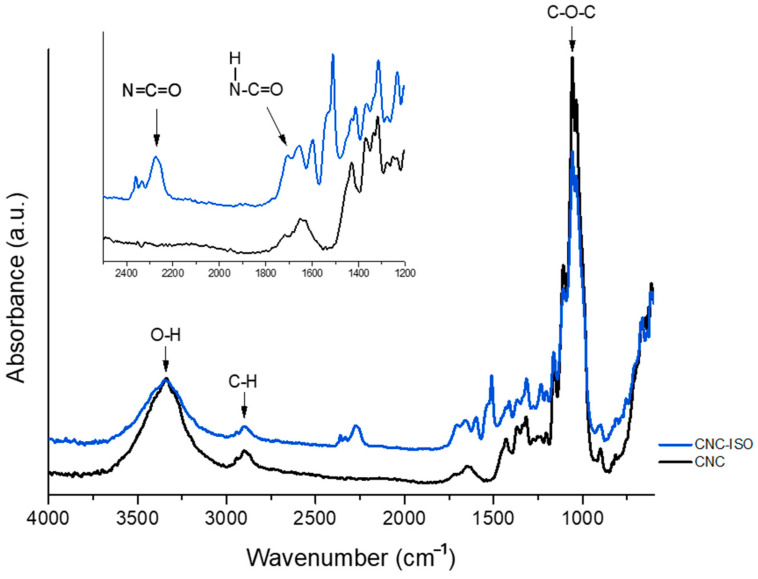
FT-IR spectra of neat CNC and ISO grafted CNC.

The FT-IR analysis was used to confirm the modification of CNC with MDI isocyanate groups and the corresponding spectra are presented in Figure 4.

Both spectra display characteristic signals to the chemical structure of CNC. The broad signal from ~3400 cm^−1^ corresponds to the stretching vibration of the hydroxyl group in polysaccharides and to the inter- and intra-molecular hydrogen bond vibrations [38,39]. The absorption peak around 2900 cm^−1^ is mainly related to C–H stretching vibrations and the signal from 1060 cm^−1^ is characteristic of the C–O–C stretching vibrations from the polysaccharide unit [40].

The successful grafting of MDI moiety onto the CNC backbone is confirmed by the appearance of a characteristic urethane bond signal near 1700 cm^−1^ [41]. Along with that, the decreased intensity of the OH (~3400 cm^−1^) and C–O–C (1060 cm^−1^) signals can be associated with the modified chemical structure of CNC. In the first case, the decreased intensity may be associated with the reaction of hydroxyl functionalities with NCO groups. In the second case, the urethane bonds formed between isocyanate and hydroxyl groups can alter the natural vibrational modes of the C–O glycosidic bonds. The presence of such urethane groups also induces steric hindrance that further restricts the vibrational freedom of C–O–C bonds. The strong polarity of urethane bonds leads to a redistribution of the electron density around the glycosidic bonds, which probably lowers dipole moment across the glycosidic linkages and results in decreased IR absorption intensity.

The absorption peak at 2250 cm^−1^ evidences the presence of the remaining free isocyanate groups on the surface of the CNC-ISO to allow further chemical interaction with the polyol component [42].

#### 3.1.2. X-Ray Photoelectron Spectroscopy Analysis (XPS) 

**Figure 5:**
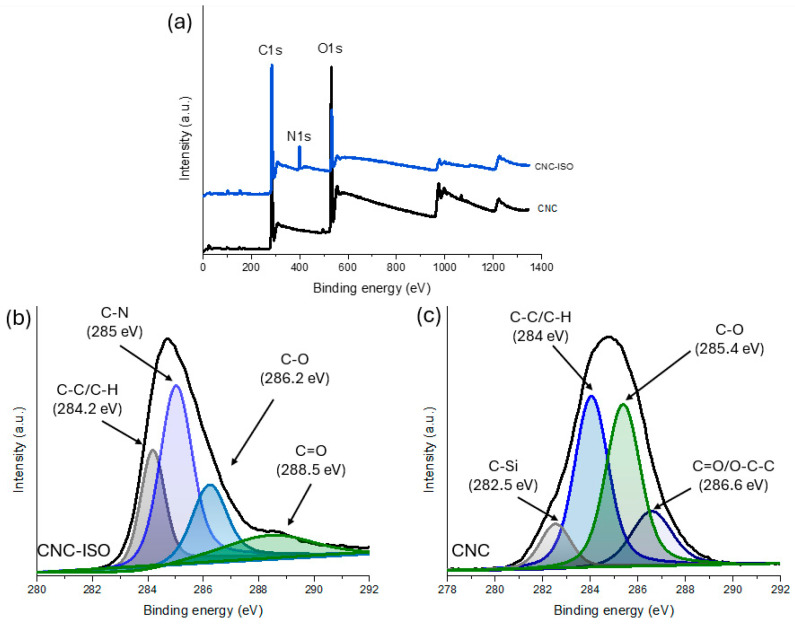
(**a**) XPS survey and C1s spectra of (**b**) CNC-ISO and (**c**) CNC.

The C1s spectrum from the XPS analysis of the CNC raw material presents the typical carbon chemical bounds found in the cellulose structure. The atomic composition of CNC and CNC-ISO, as summarized in Table 2, supports these observations. In the case of the unmodified CNC sample, the C1s spectrum evidenced the presence of the C–C/C–H bonds from the carbon backbone of the cellulose at ~284 eV [43]. Also, the presence of the C–O and C=O/O–C–O bonds from oxidized groups of cellulose such as ethers or esters were observed at 285.4 eV and 286.6 eV, respectively [44]. Additionally, a relatively lower signal was observed at 282.5 eV, which may originate from silicon impurities introduced during the processing steps. After the CNC was chemically functionalized with isocyanate, the C1s spectrum of CNC-ISO is significantly changed as new functional groups are formed. As observed from Figure 5b, the grafting of the ISO groups maintains the CNC backbone intact, indicating the C–C/C–H bonding at ~284.2 eV. However, the presence of the strong signal at 285 eV is attributed to C–N bonds [45,46], which form as a result of the reaction between isocyanate (–N=C=O) and the hydroxyl groups of CNC, producing urethane linkages (–O–C(=O)–NH–). The peak at 286.2 eV corresponds to C–O bonds, found both in the cellulose ether groups and new C–O bonds from the urethane functional group. The presence of a new peak at 288.5 eV is particularly significant, as it corresponds to the C=O bond in the urethane group, confirming the successful modification of CNC with isocyanate. Moreover, the introduction of isocyanate structures to the CNC surface is also supported by the XPS survey results as presented in Figure 5a. These observations agree with the FT-IR results, which confirmed the successful isocyanate group modification of CNC.

#### 3.1.3. Differential Scanning Calorimetry Analysis (DSC)

**Figure 6:**
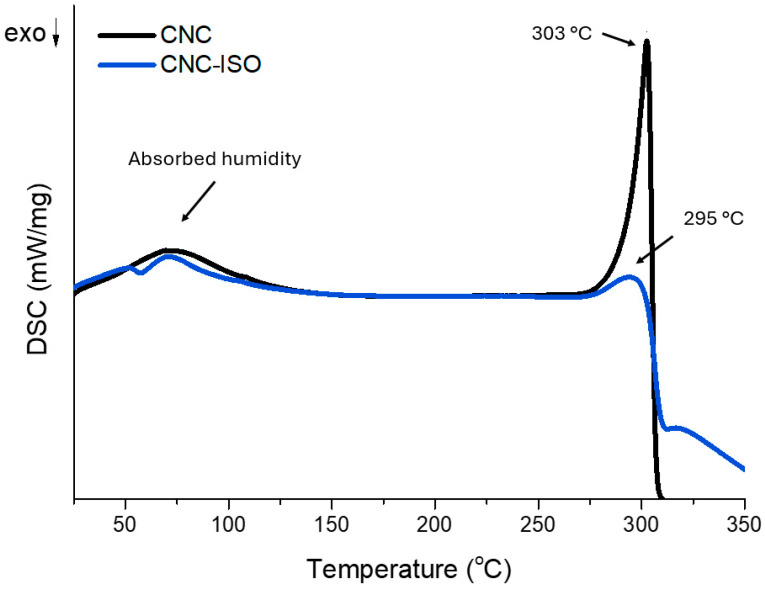
DSC spectra of pure and ISO grafted CNC.

Differential scanning calorimetry was used to assess the thermal behavior of CNC and CNC-ISO. Figure 6 displays the resulting thermograms and Table 3 shows the corresponding results. As illustrated in the DSC analysis, modification had a strong effect on the thermal properties of CNC with a large change after isocyanate modification. Both thermograms display two main transitions. The first one is associated with the loss of physically absorbed moisture and the second one with the thermal degradation of the cellulose.

The water-absorbing capacity of CNC comes from the multiple hydrophilic OH groups present on its surface. Consequently, it is expected that after chemical modification with isocyanate, the hydroxyl content to be considerably lowered. In the case of the first thermal event, the temperature rise in the T_max1_ value from 70.5 °C for unmodified CNC to 72.0 °C for CNC-ISO represents an improvement in thermal stability, which may be due to urethane linkages formed between isocyanate and hydroxyl functionalities at the surface of CNC [47,48].

Furthermore, there is a substantial decrease in ΔH_2_ (447.0 J/g for CNC to 181.4 J/g for CNC-ISO), implying that the thermal degradation of the modified CNC required less energy than the unmodified CNC. A higher T_max2_ associated with pure CNC (302.8 °C) than with CNC-ISO (294.6 °C) might be connected to the thermal stability of neat CNC in comparison to a modified one. Apart from that, the allure of the degradation peak suggests that in the case of CNC-ISO, the crystalline structure of nanocellulose is altered by the presence of the NCO moieties [49].

#### 3.1.4. Thermogravimetric Analysis

**Figure 7:**
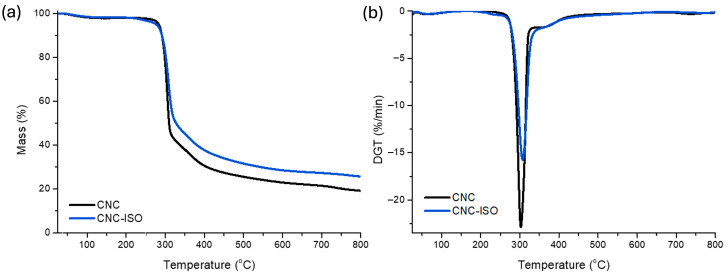
TGA (**a**) and DTG (**b**) curve of CNC and CNC-ISO.

The thermal decomposition features of CNC and CNC-ISO were also studied using the TGA. The obtained thermograms are presented in Figure 7 and the relevant thermal data are summarized in Table 4. For CNC-ISO, the initial temperatures of thermal degradation (T_d3%_ and T_d10%_) are shifted to lower temperatures than for pure CNC, which reflects a change in the initial decomposition mechanism with the introduction of isocyanate groups. On the contrary, the T_d30%_ and T_max_ of CNC-ISO are higher than those of untreated cellulose, indicating improved thermal stability. The improvement can be correlated to the formation of urethane linkages that stabilize CNC at high temperatures. Furthermore, the high percentage of residual mass, meaning high production of char, may be correlated with higher flame retardancy capacity [50].

### 3.2. CNC-Polyurea Nanocomposites Characterization

#### 3.2.1. Dynamic Mechanical Analysis (DMA)

**Figure 8:**
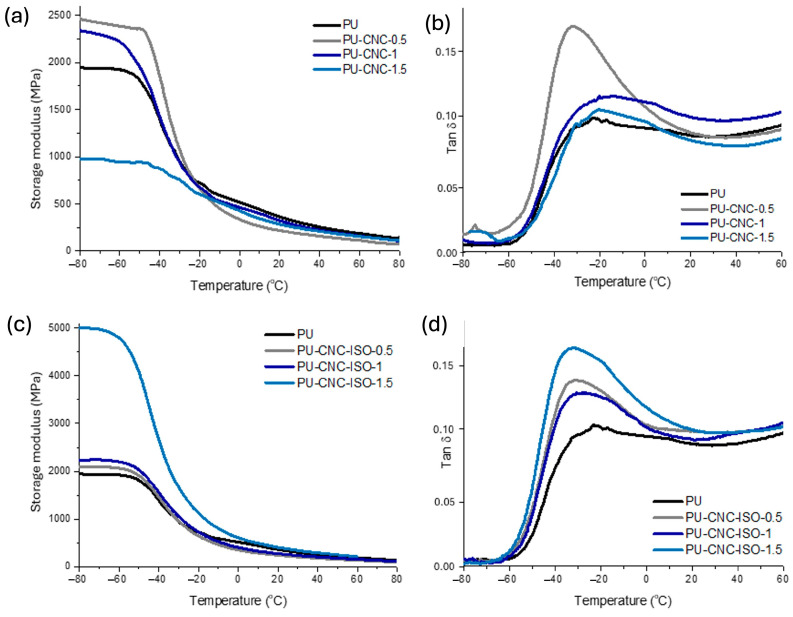
DMA curves of CNC and CNC-ISO polyurea nanocomposites: (**a**) storage modulus of PU and PU-CNC samples; (**b**) tan δ of PU and PU-CNC samples; (**c**) storage modulus of PU and PU-CNC-ISO samples; (**d**) tan δ of PU and PU-CNC-ISO samples.

The viscoelastic characteristics of CNC and CNC-ISO nanocomposites were studied by DMA. Figure 8 confirms that both the type of CNC and concentration within the formulation have a significant influence and lead to different behaviors. In the case of PU-CNC nanocomposites, the OH groups of CNC are expected to react with isocyanate groups of MDI and thus increase the overall performance of the materials. Concentrations of 0.5% and 1% CNC added to the materials increased its stiffness compared to neat polyurea, which means that CNC interacts with the polymeric matrix through hydrogen bonds. These interactions develop between the numerous OH groups present on the basal plane of CNC and urea linkage from the polyurea matrix. Nevertheless, the stiffness of nanocomposite considerably decreased at 1.5% CNC, leading to poorer mechanical performance [51,52]. This effect can be caused by the agglomeration of CNC in the matrix that destroys the integrity of the hard and soft segments in the matrix. The nanocomposites based on CNC-ISO display a different conduct. Thus, at lower loadings of 0.5% and 1%, the storage modulus is similar to that of the PU matrix, whereas PU–CNC–ISO at 1.5% loading exhibits a 2.5-fold higher modulus compared to the PU matrix. This may be due to the increased compatibility of the components owing to the isocyanate functionalization of CNC nanostructures and also due to better dispersion and interfacial bonding at high nanoreinforcing agent content [53].

The homogeneity of the system was evaluated by the shape of tan δ peaks. Upon a closer look at the tan δ curves of the PU-CNC samples, it can be observed that the allure of the tan δ signal for composite containing 0.5% CNC is narrow in comparison with the other samples. This might indicate good compatibility between the reinforcing agent and polyurea matrix, which consequently enhances mechanical behavior as also confirmed by the higher modulus value for this sample [54]. However, when looking at the intensity of the tan δ peaks, one can observe that in the case of CNC nanocomposites, the peaks corresponding with the samples with 1 and 1.5% loadings have a similar shape with the PU matrix, while in the case of CNC-ISO materials, their tan δ peaks heigh exceed the PU matrix. According to the study proposed by Bashir and collaborators [55], the height of the tan δ peaks can also provide valuable information related to the homogeneity of the system. Thus, according to these observations, the presence of CNC nanofiller within the polyurea matrix alters the polymer chain organization, affecting the homogeneity of the system. However, the improved mechanical properties may be a consequence of the formation of an extensive hydrogen bond network within the analyzed materials.

In the case of isocyanate-modified CNC polyurea nanocomposites, CNC-ISO behaved contrary to neat CNC when processed under similar conditions. Also, the tan δ peak showed that the presence of the isocyanate group on the CNC surface enhanced the compatibility of the nanoreinforcing agent with the polyurea matrix. Unlike that of PU-CNC, the 1.5% CNC-ISO sample exhibited a modulus approximately 2.5 times higher than neat PU. This finding agrees with the hypothesis that single-stage functionalization with isocyanate can provide improved coupling between system components. This not only maintains material integrity but also generates hydrogen-bonding interactions within the polymer network that lead to improved mechanical properties.

These findings show that the type of CNC used will determine their overall influence on the properties of the polyurea films. The incorporation of neat CNC might increase flexibility, while CNC-ISO may induce high rigidity and thermal stability, enabling configurable properties for specific applications.

**Figure 9:**
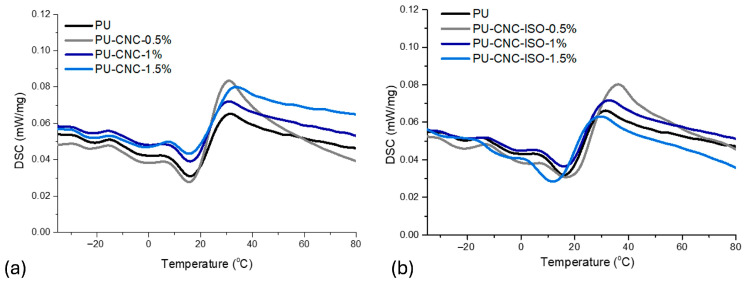
DSC thermograms of PU nanocomposites based on (**a**) CNC and (**b**) CNC-ISO.

Glass transition temperature (T_g_) represents a valuable parameter for elastomeric materials due to its strong impact on the properties. As can be seen from Table 4, all the samples showed a T_g_ below 0 °C which is also consistent with the rubbery nature of the synthesized materials. As complementary techniques to characterize the T_g_ of polyurea, DSC and DMA both provide valuable information about the networks, and the corresponding results are presented in Figure 8 and Figure 9. DSC can show microphase segregation in polyurea, unlike DMA which only indicates it via the emergence of two-step transitions. The DMA results presented in Table 4 display that along with the CNC loading increase, there are differences in T_g_, suggesting changes in chain mobility and interactions within the polyurea matrix. Thus, the lower values for the T_g_ (−26.2 °C) associated with the soft domains in the case of the PU-CNC-ISO-1.5 sample represent higher flexibility, while the highest values for the T_g_ of hard domains (18.1 °C) reflect reduced rigidity and toughness [56].

In the case of the T_g_ for the hard domains, one can observe that for CNC-ISO nanocomposites, higher values were obtained as compared with the matrix and CNC samples. This is an indicator that in this case, the nanoreinforcing agent contributes to the formation of a more compact and denser network. These results agree with the crosslinking density presented in Table 4. These parameters give valuable information about the network integrity, and it can clearly be observed that there is a significant influence of the type of CNC used over the network’s density. Thus, when neat CNC was integrated, crosslinking density decreased probably due to reduced compatibility between the polymeric matrix and the reinforcing agent used. As expected, in the case of the highest concentration of CNC (1.5%), the tendency to agglomerate increases, and thus, the possible interactions that may additionally form between the components of the system are reduced. The superior values determined for the CD of CNC-ISO nanocomposites confirm the formation of strong interfacial interactions. In this case, additional interactions are developed between the components of the systems and these interactions proportionally increase with the filler content.

#### 3.2.2. Thermo-Gravimetrical Analysis (TGA)

**Figure 10:**
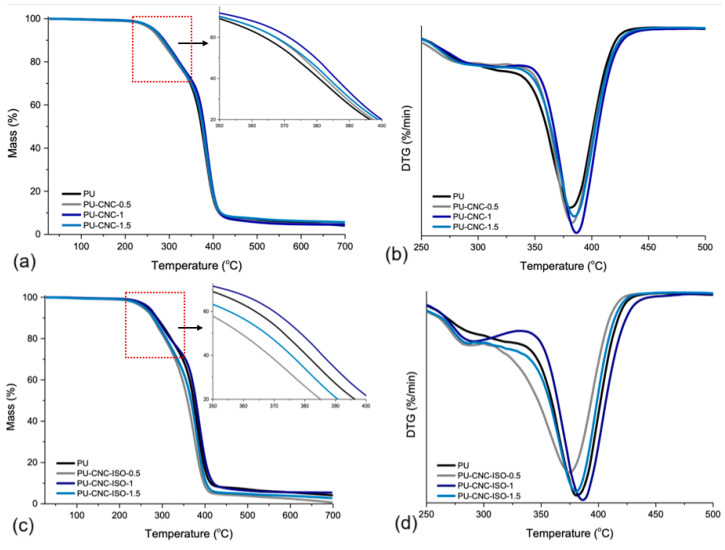
TGA (**a**,**c**) and DTG (**b**,**d**) curves of CNC and CNC-ISO polyurea nanocomposites.

The impact of the CNC type and concentration on the thermal stability of polyurea composites was evaluated by TGA and the corresponding thermograms are presented in Figure 10. The thermal data presented in Table 5 show different degradation behavior between CNC and CNC-ISO. With respect to directly integrated CNC series (PU-CNC), the T_d3%_ of PU-CNC-0.5 decreased slightly by 5% compared to neat PU. This could mean a slight destabilization of the polymeric matrix through direct CNC interaction. But when the CNC contents were higher, the degradation temperatures moved to a higher temperature and the residue mass increased too, which may indicate that CNC could have a reinforcement effect at a high content.

However, at higher CNC-ISO loadings, the degradation temperatures are comparable to or somewhat higher than those of pristine PU, probably due to a more thermally stable internal network in the polyurea phase. The presence of the lowest weight loss at 1.5% of CNC-ISO was clearly lower than that found for the direct CNC series, which could reflect a different mechanism in relation to structural changes or even a char-forming residue after degradation [57].

Additionally, the T_max_ generally increases with CNC addition, which could be attributed to increased thermal stability due to the dispersion of CNC and CNC-ISO within the matrix. This means a more complex equilibrium between CNC or CNC-ISO surface loading and the thermal stability of the PU.

The 0.5 wt% CNC-ISO formulation exhibits the lowest onset-degradation temperatures (T_d3%_ = 239 °C; T_d10%_ = 277 °C), the lowest T_max_ (374 °C), and the smallest char yield (0.65 %) in Table 5. At this loading, the grafted nanofiller is below the percolation level required to build a continuous, char-forming network. On the contrary, it partially disrupts the original polyurea hydrogen bond/internal network, leading to the lowest crosslinking density (5.44 mol cm^−3^, Table 4). Along with the increase in CNC-ISO nanofiller content to 1.0–1.5 wt%, covalent urethane interphases become sufficiently dense; thus, T_d_ and T_max_ slightly exceed the neat PU values. The pronounced deviation of PU-CNC-ISO-0.5 is, therefore, a concentration effect inherent to the early stage of network formation.

### 3.3. Mechanical Analysis

**Figure 11:**
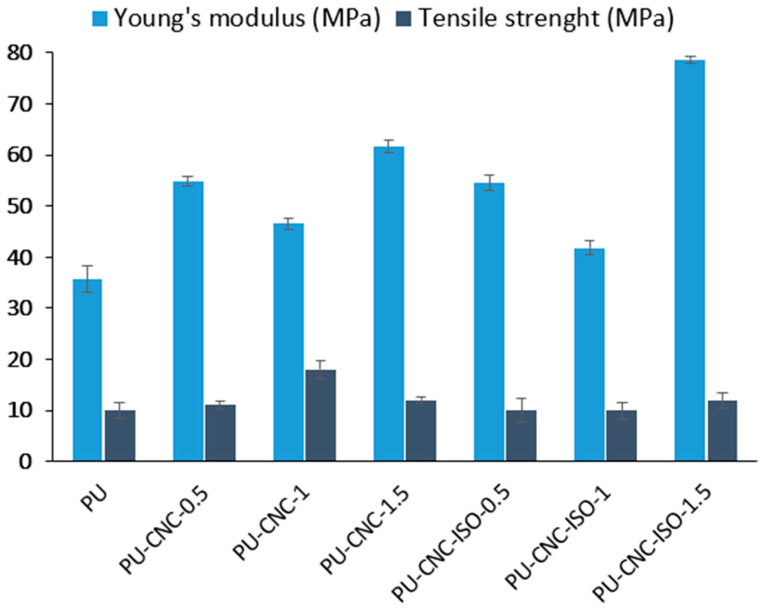
Tensile mechanical properties of neat and CNC-reinforced polyurea nanocomposites.

Tensile strength and Young’s modulus were obtained from the same un-axial tests performed on an Instron 3382 at 1 mm min^−1^ in accordance with EN ISO 527-3 and the mean values are shown in Figure 11.

Neat CNC increases tensile strength only at 1 wt% (PU-CNC-1). At this loading, the crystals are still individually dispersed and can transfer load to the matrix through hydrogen bonding, giving a ~16 % gain over PU. Below this level (0.5 wt%), the filler population is insufficient to form a load-sharing network, while above it (1.5 wt%), excess hydroxyl groups promote CNC–CNC association; the resulting aggregates act as stress concentrators and the strength returns to the PU baseline, a trend also reported for other CNC-filled polymers [58].

Every composite is stiffer than neat PU, yet the magnitude and the associated loss of ductility depend on filler chemistry and amount (Figure 11, Table 4). The modest drop in modulus seen at the intermediate loading (1 wt%) for both series coincides with an increase in elongation at break. This response is attributed to internal plasticization: interactions between the nanofiller surface and the soft segments disturb the original hydrogen bond array, lower the microphase transition temperature, and allow greater segmental mobility. The enhanced mobility permits limited reorientation of hard domains under low strain, which can raise the initial elastic slope even as the overall network momentarily softens—a mechanism previously described for PEI-plasticized polyurethanes [59].

At 1.5 wt% loading, the two fillers diverge sharply. CNC-ISO carries pendant –NCO groups that react with amine or residual –OH sites during curing, creating covalent urethane bridges between the nanocrystals and the polymer chains. Each crystal thus acts as a multifunctional crosslinker, raising the calculated network density from 5.05 mol cm^−3^ for PU-CNC-1.5 to 6.27 mol cm^−3^ for PU-CNC-ISO-1.5 (Table 4) and boosting the modulus to ~80 MPa, i.e., ≈128% above neat PU. This large gain is consistent with earlier reports that surface-reactive fillers generate more effective load-bearing interfaces than untreated nanocellulose in polyurethanes [60] and with related studies on IPDI-grafted CNC [28] and polyol-functionalized CNC [50].

The 128% improvement in modulus observed with the addition of CNC-ISO-1.5% highlights the reinforcing efficiency of the filler, but it also introduces notable trade-offs in processability. Increased CNC loading typically results in significantly higher viscosity of the polyurea matrix, making the material more difficult to mix, cast, or inject using standard processing methods. Furthermore, achieving uniform dispersion of CNC-ISO becomes more challenging at higher concentrations, often leading to agglomeration, which can compromise both mechanical performance and reproducibility. These effects may require additional processing steps such as ultrasonication, high-shear mixing, or the use of surfactants or compatibilizers to maintain composite homogeneity. Thus, while the enhancement in stiffness is desirable, it must be balanced against the increased complexity, cost, and potential limitations in manufacturing scalability.

In contrast, neat CNC relies solely on hydrogen bonding. At 1.5 wt%, these interactions promote particle–particle clustering rather than particle–matrix bonding. The resulting micro-voids disturb the hard/soft segmentation of the polymer and restrict effective stress transfer, so the modulus plateaus at ~60 MPa, well below the CNC-ISO analog.

The large modulus gain for CNC-ISO was anticipated because surface-reactive particles produce covalent load-bearing bridges that stiffen segmented polyurethane matrices more efficiently than untreated fillers [56]. Similar “reactive-filler” behavior has been reported for IPDI-grafted CNC in elastomers [28] and polyol-functionalized CNC in PU foams [50].

#### Scanning Electron Microscopy Results

**Figure 12:**
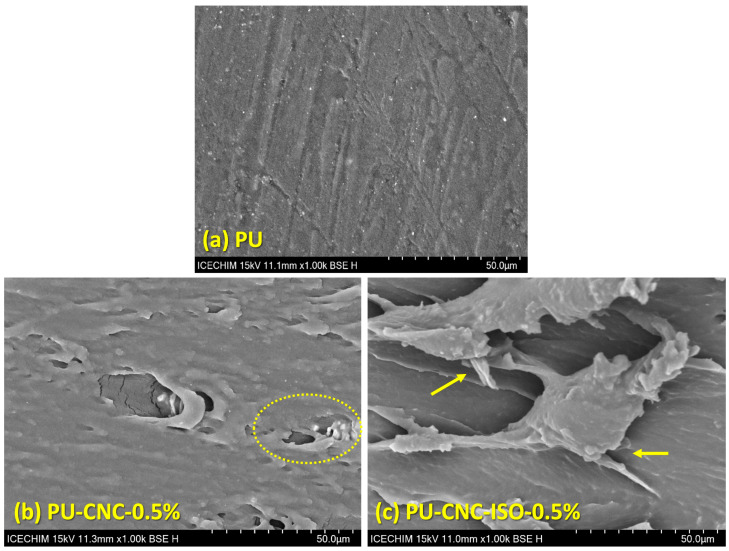

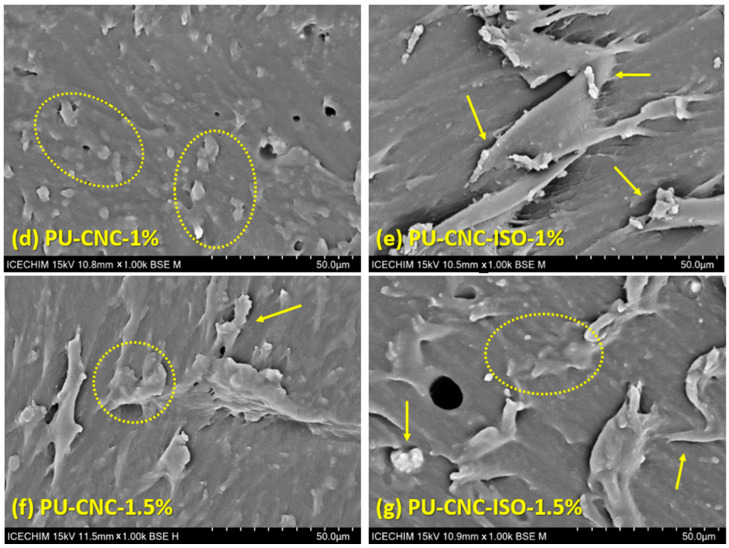
SEM micrographs of polyurea-based nanocomposites.

Compatibility and dispersion between CNC and polyurea matrix were examined using an SEM analysis. As depicted in Figure 12, the morphology of the neat PU displays a smooth surface. The structural defects such as holes observed in the micrographs of the nanocomposites appear as a consequence of the localized heating induced by the electron beam which can cause the softening, melting, or decomposition of temperature-sensitive polymers [61].

For the PU-CNC samples, along with the increase in the CNC content, the fracture surface becomes rough with more heterogeneity. This may be related to the agglomeration of CNC particles or certain dispersion deficits in the polyurea matrix, therefore causing morphological change.

The surface morphology of the PU-CNC-ISO nanocomposites exhibits a distinct interface with the PU matrix. The introduction of CNC-ISO into the polyurea matrix resulted in a more irregular texture at the fracture surface, which is characteristic of enhanced interfacial bonding between the polymeric matrix and the nanofiller. Parallel lines formed on the fracture surface reveal the direction of fracture propagation, which can be considered as another piece of evidence resulting from the presence of nanocellulose structures with high rupture resistance resulting from the presence of nanocellulose structures that resisted rupture. These findings indicate the successful achievement of interfacial compatibility of the CNC-ISO within the PU matrix, even at 1.5% of incorporating content.

At the same time, in the case of the functionalized CNC nanocomposite samples, a pull-through effect can be observed. This demonstrates that the CNC-ISO facilitates energy dissipation during fracture by introducing a more complex and irregular fracture, resulting in improved strength and mechanical properties of the nanocomposites [62,63].

The SEM micrographs display that crack propagation in neat CNC/polyurea is governed by matrix-dominated failure and CNC pull-out because stress is transferred mainly through secondary hydrogen bonding at a relatively weak interface [28,53]. Grafting CNC with MDI (CNC-ISO) introduces covalent urethane linkages, greatly increasing interfacial shear strength. The result is a tortuous, fibrillated fracture surface with “pull-through” ridges that signal crack bridging and energy dissipation by the debonding of well-anchored CNC [48,55]. The stronger interface promotes more efficient load sharing, reflected in the ~128 % rise in modulus and delayed crack growth reported for PU-CNC-ISO-1.5 wt% [56]. Thus, functionalization transforms the interface from a slip plane into an active load-bearing network, dictating the observed fracture morphology and the superior mechanical performance of the CNC-ISO nanocomposites.

## 4. Conclusions

The spectroscopic analyses confirmed that grafting isocyanate onto cellulose nanocrystals (CNC-ISOs) forms covalent urethane linkages within the polyurea matrix. This is evidenced by a new C=O band at approximately 1700 cm^−1^ and a C–N signal at 285 eV (about 7 at% N 1s). This chemical anchoring enhances the dispersion of the nanofiller and integrates the CNC into the network structure.

Incorporating 1.5 wt% CNC-ISO increases the crosslink density from 5.05 to 6.27 mol/cm^3^. Dynamic mechanical testing reflects this change, as the storage modulus increases 2.5 times, and the soft segment’s glass transition temperature (T_g_) shifts to −26 °C, indicating a stiffer and more uniformly phased material.

These molecular improvements result in notable enhancements at the macroscale. The same loading of CNC-ISO elevates Young’s modulus to approximately 80 MPa (+128% compared to neat polyurea) and increases tensile strength by 29% relative to the neat CNC analog. Scanning electron fractography corroborates these enhancements, revealing dense, fibrillated crack bridges instead of the void-rich fracture surfaces observed in unmodified films.

Thermal performance follows a similar trend: the primary decomposition peak shifts to approximately 380 °C, and the char yield doubles, indicating that the covalent interface enhances the network’s stiffness and creates a more effective protective char layer.

Processing remains practical, requiring only a four-hour grafting step and mixing at 80 °C. This results in a viscosity increase from 1.2 to 3.8 Pa·s, which fits comfortably within standard casting protocols. Since CNC is derived from renewable biomass, CNC-ISO composites combine these performance improvements with a reduced reliance on purely petrochemical reinforcements, offering a more sustainable approach to producing high-performance polyurea materials.

In summary, a modest loading of CNC-ISO results in a thermally and mechanically superior—and inherently more sustainable—polyurea material while maintaining manufacturing practicality. This makes CNC-ISO a promising additive for protective coatings and other high-performance applications.

## Figures and Tables

**Table 1:** Nanocomposite samples composition description and abbreviations.

Nr.crt	Part A			Part B	Polyurea Sample Abbreviation
	CNC Type in the Prepolymer Formulation	CNC Amount, wt%	Polyol	Amine	
1	Blank prepolymer	0	D-2061	D2000-E300	PU
2	CNC	0.5	D-2061	D2000-E300	PU-CNC-0.5
3	CNC	1	D-2061	D2000-E300	PU-CNC-1
4	CNC	1.5	D-2061	D2000-E300	PU-CNC-1.5
5	CNC-ISO	0.5	D-2061	D2000-E300	PU-CNC-ISO-0.5
6	CNC-ISO	1	D-2061	D2000-E300	PU-CNC-ISO-1
7	CNC-ISO	1.5	D-2061	D2000-E300	PU-CNC-ISO-1.5

**Table 2:** Atomic percentages of pure CNC and isocyanate-grafted CNC (CNC-ISO) from XPS results.

	C1s (Atomic %)	O1s (Atomic %)	N1s (Atomic %)
CNC	61.84	38.16	-
CNC-ISO	76.90	13.63	6.92

**Table 3:** DSC and TGA data for CNC and CNC-ISO.

Sample	ΔH_1_ (J/g)	T_max1_ (°C)	ΔH_2_ (J/g)	T_max2_ (°C)	T_d3%_ (°C)	T_d10%_ (°C)	T_max_ (°C) from DTG	Residual Mass (%)
CNC	44.52	70.5	447.0	302.8	269.6	291.3	302.2	19.17
CNC-ISO	30.79	72.0	181.4	294.6	243.3	276.6	306.5	25.71

**Table 4:** Comparative glass transition temperatures (T_g_) of polyurea nanocomposites with CNC and CNC-ISO from DMA and DSC analysis and network parameters.

Sample	T_g_ (°C) from DMA	T_g_ Soft (°C) from DSC	T_g_ Hard (°C) from DSC	SD (%)	CD * mol/cm^3^
PU	−24.1	−23.6	13.6	23.80	5.71
PU-CNC-0.5	−31.6	−23.9	12.1	27.93	5.25
PU-CNC-1	−19.9	−23.5	14.2	27.58	5.28
PU-CNC-1.5	−19.5	−24.1	13.5	29.84	5.05
PU-CNC-ISO-0.5	−31.1	−23.8	14.9	26.09	5.44
PU-CNC-ISO-1	−29.8	−22.9	14.3	19.78	6.22
PU-CNC-ISO-1.5	−33.3	−26.2	18.1	19.44	6.27

*—calculated from swelling degree.

**Table 5:** Thermal degradation characteristics of CNC and CNC-ISO polyurea nanocomposites.

Sample	T_d3%_ (°C)	T_d10%_ (°C)	Residual Mass (%)	T_max_ (°C) from DTG
PU	248.7	287.7	4.06	381.4
PU-CNC-0.5	244.3	281.1	4.69	382.8
PU-CNC-1	250.2	288.6	4.49	386.6
PU-CNC-1.5	250.0	286.3	5.68	384.5
PU-CNC-ISO-0.5	239.2	277.2	0.65	373.6
PU-CNC-ISO-1	250.0	285.3	5.07	384.5
PU-CNC-ISO-1.5	244.3	280.0	2.72	380.0

## Data Availability

The data presented in this study are available on request from the corresponding author. The data are not publicly available due to institutional restrictions.
